# Biodegradation of naphthalene using Pseudomonas aeruginosa by up flow anoxic–aerobic continuous flow combined bioreactor

**DOI:** 10.1186/s40201-015-0175-1

**Published:** 2015-03-26

**Authors:** Behrooz Karimi, Maryam Habibi, Mehry Esvand

**Affiliations:** Department of Environmental Health Engineering, School of Health, Arak University of Medical Sciences, Arak, Iran; Department of Environmental Health Engineering, School of Health, Tehran University of Medical Sciences, Tehran, Iran

**Keywords:** Anoxic–aerobic continuous flow combined bioreactor, Biodegradation, Naphthalene, Pseudomonas aeruginosa

## Abstract

**Background:**

Naphthalene is a poly aromatic hydrocarbon (PAH) present in many sediment-water systems. The aim of this study was to evaluate the applicability of an anoxic/aerobic system for the biological treatment of water polluted by naphthalene by Pseudomonas aeruginosa PTCC 1707 to utilize naphthalene. The naphthalene elimination from wastewater was determined in anoxic–aerobic continuous flow combined bioreactor under continuously oxic and anoxic conditions. Experiments were conducted in continues mode, and naphthalene was administered in consecutive spike doses. Then Pseudomonas aeruginosa bacteria suspension with a specific turbidity (0.5-10 NTU) was prepared from growing bacteria on R_2_A medium and injected to reactor.

**Findings:**

At naphthalene concentration = 0.5-20 mg/L, 33–65.5% naphthalene removal efficiencies were observed. Mean COD removal efficiency in solid retention times of 2, 4, 6, and 8 days was 82.7, 92.45, 95.97 and 96.1%, respectively. Naphthalene removal efficiency by bacterium pseudomonas at pH 8 was 96% and at pH 4, 5.5, 7 and 9.5, 68, 80, 90 and 85%, respectively. As the initial concentration of naphthalene increased from 0.5 to 20 mg/L, the remaining concentration of naphthalene decreased from 33.4% to 65.5% after 3 days.

**Conclusion:**

Based on experimental results, it was determined that this process can effectively reduce naphthalene under optimal conditions and this method can be used for the removal of similar compounds.

## Introduction

Remediation of contaminated soil and groundwater is an important issue that is of concern among environmentalists and hydrogeologists [1]. Polycyclic aromatics hydrocarbons (PAHs, also known as polycyclic organic matter or POM) are chemical species with two to six fused benzene rings and are well known toxic hazardous pollutants and highly potent carcinogens that can cause tumors in some organisms [2]. PAHs originate from natural and anthropogenic sources. Anthropogenic sources include engine exhaust, industrial processes, crude oil, urban run-off, domestic heating systems, incinerators and smoke. Natural sources include terrestrial coal deposits, volcanic eruptions and forest fires. The main sources of PAHs in surface water are atmospheric deposition, run-off from contaminated soils and deposition from sewage discharges [3]. The main characterized of PAHs were high toxicity, concentrated substrate and salt (usually exceeding 3 wt.%), and poor decolorization and biodegradation.

Naphthalene is the simplest, most volatile and least toxic of the PAHs. In fact naphthalene has been used in several research laboratories as a model to develop catalysts and biological process with potential to effectively destroy PAHs [4]. Albeit naphthalene has a relative low solubility in water (32 mg/L) but it is highly hazardous. About 5% of all naphthalene disposed into the environment is released into water. Studies of naphthalene degradation may be significant because naphthalene is a common pollutant that serves as a chemical model for the degradation of PAHs [5]. The range of naphthalene concentration in wastewaters is from ng/L to mg/L. Concentrations such as 1.65 mg/L were reported on wastewater samples from the radioisotope manufacturing facilities, 6–220 ng/L, in municipal wastewater, 285 mg/L (as naphthalene sulfonic acid), in effluents from ion-exchange resin towers, and 0.1–2.1 mg/L in dyeing and textile wastewater [6].

Various physical, chemical, biological, and their combined technologies have been attempted to remediate organic-contaminated waters. The in situ microbial degradation of PAHs is limited by their low bioavailability and low water-solubility [7]. Among various attempts made to treat such wastewater, such as, evaporation, polymeric absorption, solvent extraction, and conventional biological treatment, are proved ineffective due to the quite stable structure of the naphthalene ring and the high concentrations of salt and acid in the streams from the dye manufacturing process [8]. The methods for treating PAHs mainly include biodegradation, scrubber absorption, high-energy electron beam irradiation, ozonation, catalytic combustion volatilization, photo-oxidation, chemical oxidation and adsorption [9]. Most of the PAHs such as naphthalene are not aerobically degraded in activated sludge system, as the benzene rings is susceptible to reduction under low redox potential. The oxic/anoxic (O/A) system is an alternative to the traditional activated sludge process for treating high naphthalene solution [10].

Recently, a number of oxic/anoxic bacteria have been used to biodegrade naphthalene with several pathways and metabolic diversities described [11]. Bacteria such as Pseudomonas putida, Rhodococcusopacus, Mycobacterium sp., Nocardia otitidiscaviarum, and Bacillus pumilus have been reported to biodegrade naphthalene. Pseudomonas aeruginosa is an environmental bacterium that can be isolated from many different habitats, including water, soil, and plants, but it is also an opportunistic human pathogen causing serious nosocomial infections [12]. Pseudomonads are the best-known bacteria capable of utilizing hydrocarbons as carbon and energy sources and producing bio surfactants which enhance the uptake of such immiscible hydrophobic compounds.

The aim of this study was to evaluate the applicability of an anoxic/aerobic system for the biological treatment of naphthalene by P. aeruginosa to utilize hydrocarbons ranging from naphthalene to polycyclic aromatic hydrocarbons as a carbon source. Furthermore, P. aeruginosa growth conditions in anoxic-aerobic reactor, that constitute the most important factor affecting biodegrading efficiency, kinetics, and the biodegradation mechanism of naphthalene, have been investigated.

## Methods

### Chemical

Naphthalene (99.9% purity), was supplied by MERCK. The physicochemical properties of naphthalene used in this study are listed in Table 1. R_2_A agar, Murashige and Skoog Basal Salt Mixture (MS salts), Benzyl-adenine and Indole-3-acetic acid (IAA) were purchased from Sigma–Aldrich (USA).

**Table 1:** **Physico**-**chemical properties of naphthalene**

Molecular weight	128.19
Formula	C_10_H_8_
Boiling point	218C
Melting point	80.5C
Solubility (at 20°C)	32 mg/l
Specific gravity	1.145
Henry’s law constant	20 atmm^3^ water/m^3^ air
K_ow_	2800

A minimal medium (MM) was composed of (L): KH_2_PO_4_ 1.0 g, NaCl 5 g (NH_4_) _2_SO_4_ 0.3 g, MgSO_4_ · 7H_2_O 0.3 g, CaCl_2_ 20 mg, Sugar (2.5 g/L). MM also contained trace elements as follows: (L): ZnSO_4_ 5.0 g, FeCl_3_ 2.3 g, MnSO_4_ 5.0 g, and (NH_4_) 6Mo_7_O_24_ 1.0 g. Solid MM plate was composed of (L MM): 20 g agar. Solid LB plate was composed of (L): 5 g NaCl, 10 g peptone, 5 g yeast extract, 20 g agar. Flasks containing the medium were sterilized by autoclaving at 121°C for 20 min. The pH of the medium was between 6.8 and 7.2. All chemicals used were of analytical grade and all reagents were used as supplied.

### Bacterial enumeration

**Figure 1:**
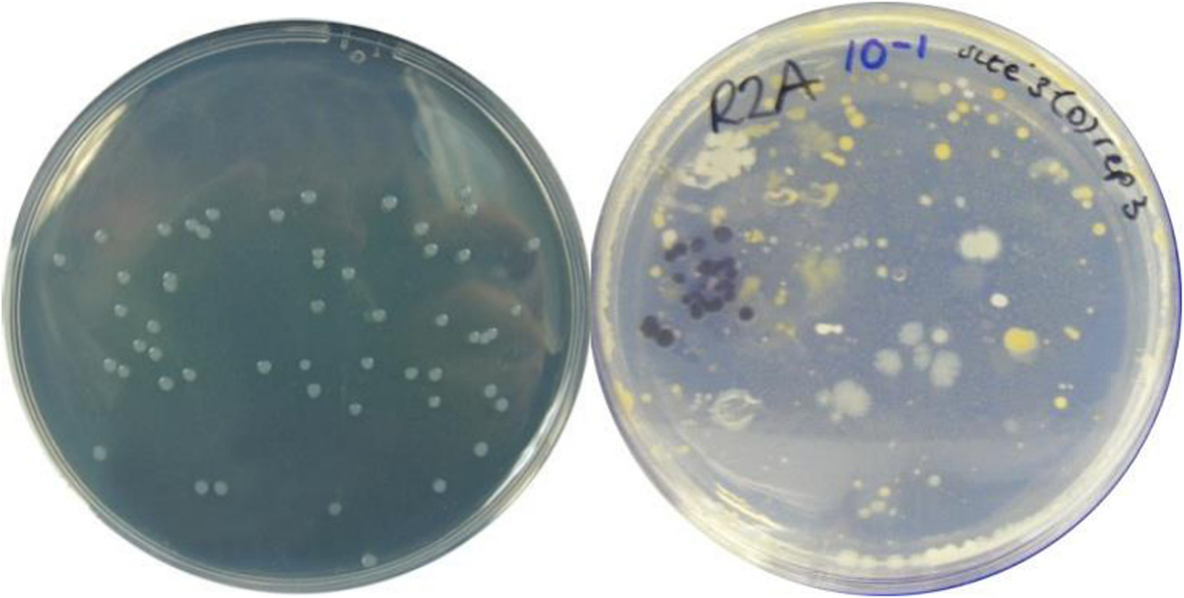
**R**_**2**_ **A medium was developed to growing the Pseudomonas aeruginosa.**

Pseudomonas aeruginosa was counted on R_2_A medium (Figure 1) and MSM containing 30 mg/L naphthalene, according to Standard Plate Count Method. Pseudomonas aeruginosa bacteria PTCC 1707 growing on the above medium (with naphthalene and Sugar as the only carbon source) were defined as naphthalene degrading bacteria. Then bacterial suspension with a specific turbidity (0.5-10 NTU) was prepared from growing bacteria.

### Synthetic wastewater

Ammonium and nitrite were supplemented to mineral medium as required in the form of (NH_4_)_2_SO_4_ and NaNO_2_, respectively. The composition of the mineral medium was (g/L except for trace element solution): KH_2_PO_4_ 0.01, CaC_l2_.2H_2_O 0.00565, MgSO_4_.7H_2_O 0.3, KHCO_3_1.25, FeSO_4_ 0.00625, EDTA 0.00625 and 1.25 mL/L of trace elements solution. The trace element solution contained (g/L): EDTA 15, H_3_BO_4_ 0.014, MnCl_2_.4H_2_O 0.99, CuSO_4_.5H_2_O0.25, ZnSO4.7H_2_O 0.43, NiCl_2_.6H_2_O 0.19, NaSeO_4_.10H_2_O 0.21, NaMoO_4_.2H_2_O 0.22 and NaWO_4_.2H_2_O 0.050. Synthetic wastewater containing naphthalene was gradually entered into the reactor. Naphthalene was added to deionized water and stirred for 12–14 h to prepare a saturated solution. The naphthalene with concentrations 0.5-20 mg/L was prepared and introduced in reactor. The pH was adjusted using either concentrated sulfuric acid or 0.25 N NaOH.

### Biodegradation experiments

Various factors were examined for their influence on growth of selected strain and on the biodegradation of naphthalene. These included temperature, pH, nitrogen concentration, salinity, inoculum concentration, different naphthalene concentration and SCOD. Various conditions are briefly described: incubation temperatures of 20, 25, 30, 35 and 40°C; autoclaved medium pH of 5.5, 6.0, 6.5, 7.0, 7.5, 8.0 and 9.0 using 1 M HCl or 1 M NaOH; the C/N ratio of 0.7, 1.0, 1.3, 2.0, 4.0 and 20.0 using naphthalene and (NH_4_)_2_SO_4_; the salinity of 0, 1, 5, 8, 10, 15 g/L with NaCl [11] and naphthalene at concentrations of 0.5, 1, 5, 10 and 20 mg/L were used for this experiment. The cultures were incubated in a shaking incubator at 150 rpm, 30°C. The naphthalene concentration after degraded in various samples was determined using gas chromatography. All experiments were carried out in triplicate. The degree of naphthalene degradation was calculated as:

$$ \mathrm{Degradation}\ \mathrm{efficiency}\ \left(\%\right) = \frac{C_0-{C}_t}{C_0}\times 100 $$

Where C_0_ and C_t_ stand for the naphthalene concentrations before and after degradation, respectively.

### Reactor systems and experimental conditions

**Figure 2:**
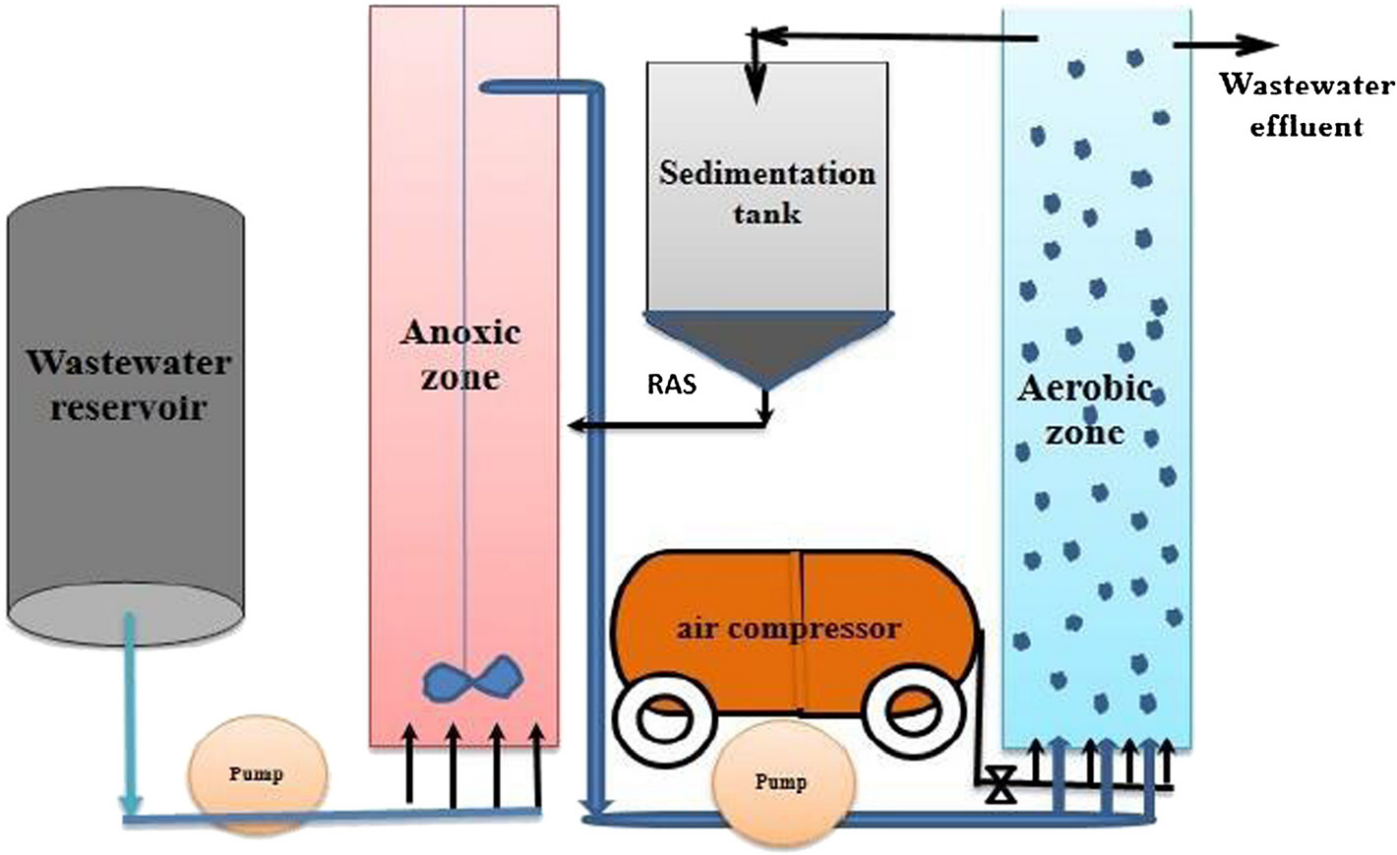
**Schematic of the laboratory reactor system.**

The experimental system employed in this study consisted of one reactor with two stages (anoxic and oxic) with a working volume of five liters in continues conditions with stirring (350 rpm), have a diameter and height of 10 and 80 cm, respectively. A schematic of the laboratory reactor system is provided in Figure 2.

After 4–6 h of incubation, turbidity 0.5(1.5 × 10^6^ bacteria per ml), 1, 2, 5 and 10 NTU of the bacterial suspension (Pseudomonas aeruginosa) was obtained. The 20 mL of bacterial suspension was added per 1 L of synthetic wastewater. Reactors were filled with synthetic wastewater and bacterial suspension as biomass, with a useful volume of 5 L (Table 2). One dual-channel peristaltic pump was used to initially feed the reactors with a constant flow (0.28 L d^−1^). The influent originated from container, was stored at 4°C during the entire process. The reactor’s recycle flows were made by a peristaltic pump at a maximum flow rate of 0.55 L d^−1^. The temperature was set at 35°C and the influent pH was controlled in the range of 6.8-7.0.

**Table 2:** **Operation conditions of the anoxic and oxic reactors**

	**Anoxic zone**	**Aerobic zone**	**total**
Volume reactor	2.5 L	2.5 L	5 L
Upflow velocity (mh^−1^)	1.00	1.00	1.00
Flow rate (Ld^−1^)	0.28	0.28	0.28
Total COD (mgL^−1^)	300	300	300
HRT (h)	24	24	24
pH_influent_	4-8	4-8	4-8

The anoxic condition was obtained when oxygen concentration reached 0mgL^−1^. Aeration in the reactor was provided by filtered air using bubble diffusers. Oxygen concentrations were checked using oxygen probes maintained in the slurry throughout the experiment in all reactors. The temperature range during incubation was 20–27°C, pH was 6.4–7.7, and oxygen concentrations were over 2 mg L^−1^ in oxic section and 0 mg L^−1^ in the anoxic section. Samples were taken in triplicate for chemical analyses at 0, 2, 4, 6, 8, 12, 15, 20 and 23 days of incubation. Samples for GC analysis were withdrawn at the end of each experiment.

### GC method for analysis

The sample was added into a 40 mL glass amber vial containing 9 mL nanopure water and 10 mL acetate buffer (0.1 M at pH 5.0). 50 L sodium tetra ethyl borate (NaBEt_4_, 5%) and 20 L tetra butyltin (TeBT at 1 mg L^−1^, as an internal standard) were then added. The vial was immediately placed on a magnetic stirrer to maintain a constant agitation. A SPME fiber coated with 30 m thickness poly dimethyl siloxane (PDMS) purchased from Supelco (Bellefonte, PA, USA) was exposed to the headspace over the vigorously stirred sample for 20 min at room temperature. The fiber was then placed in the GC injector, desorbed at 250°C for 3 min.

Themeasurements ofnaphthalenewere detailed inourpreviousstudy. Naphthalene was quantified using a Varian Model 3380 gas chromatograph equipped with a flame ionization detector, Chrompack capillary column (Select 624 CB D_f_ 1.8 μm, FS 30 × 0.32 mm ID), and an auto-sampler with a 100 μm PDMS coated SPME fiber assembly (Supelco, Bellefonte, PA).

The sample adsorption time with the SPME fiber was 10 min in agitate mode and desorption time was 2 min followed by a 1 min waiting period. The analysis was performed in split less mode with an injection temperature of 250°C, isothermal oven temperature of 180°C, and detector temperature of 275°C.

The data presented are the mean values ofthree measurements with the relative standard deviations of 6.7–10.2%. The pH was measured using an Ag/AgCl combination pH electrode attached to a pH meter (Ion Analyzer 220, Corning, Inc., Corning, NY).

### Chemical oxygen demand (COD)

COD was measured in the supernatant fraction (soluble COD, CODs) according to the standard methods. By difference between COD and CODs, the particulated COD (CODp) was calculated. The COD solubilization (SCOD) represents the transfer of COD from the particulated fraction of the sludge to the soluble fraction. SCOD was calculated by using the difference between CODs after ultrasonic treatment and the initial CODs (CODs_0_) in relation to the initial particulated COD (CODp):

$$ \mathrm{SCOD}\ \left(\%\right) = \frac{\left(CO{D}_s-CO{D}_{S0}\right)}{\left(CO{D}_p\right)}\times 100 $$

The aimed total COD in the influents of reactor was 300 mg COD/L.

### Statistical analyses

Mean and standard deviation of the triplicates in each treatment are calculated. The differences among three nutrient groups were evaluated firstly by a parametric one-way analysis of variance (ANOVA). If significant difference was found at p ≤ 0.05, multiple comparison test of Tukey–Kramer was employed. All statistical analyses were performed using SPSS ver 16.

## Results and discussion

Initially, it was worked on the compatibility between bacterium pseudomonas aeruginosa and naphthalene for 3 months. For this purpose, glucose with 100 mg/L COD was used as the carbon source. In the next step, concentration of the input glucose to the system was gradually reduced, while increasing naphthalene dose. This course continued to the point where the bacterium became perfectly compatible with naphthalene, and in presence of naphthalene, COD concentration was brought within range of 30–40 mg/L.

### Growth P. aeruginosa on anoxic–aerobic reactor

**Figure 3:**
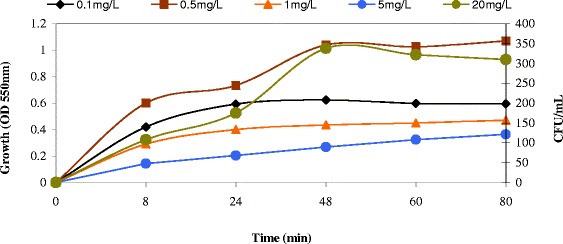
**Growth of P. aeruginosa** **(OD and CFU/** **mL)** **in vertical anoxic–** **aerobic continuous flow combined bioreactor with 0.1,** **0.5,** **1,** **5,** **10 and 20 mg/** **L for naphthalene.**

To examine the growth of P. aeruginosa (OD and CFU/mL) in vertical anoxic–aerobic continuous flow combined bioreactor and tolerance of naphthalene by P. aeruginosa, the cells were cultivated in nutrient broth with concentration of naphthalene. It appears that the cells were able to survive naphthalene concentration as high as 20 mg/L naphthalene. Figure 3 shows the growth curves of P. aeruginosaAT18 in the presence of naphthalene, this can be attributed to growth of biomass on naphthalene intermediates. Goel et al. [13] reported a similar observation of growth of naphthalene degrading culture on their catabolic intermediates.

### Effect of retention times on naphthalene degradation during anoxic–aerobic process

**Figure 4:**
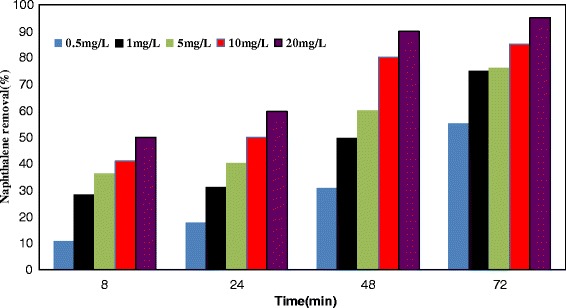
**Effect of bacterial concentration on naphthalene degradation in hydraulic retention time of 20–** **80 minutes and in cellular retention time of 2–** **8 days.**

**Figure 5:**
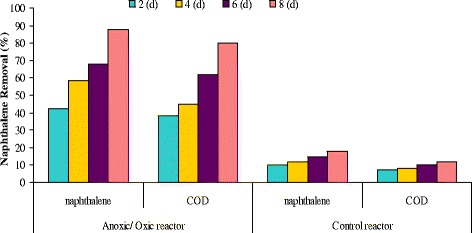
**Naphthalene and COD removal from water by bacterium pseudomonas aeruginosa in vertical anoxic–** **aerobic continuous flow combined bioreactor and control reactor at cellular retention time 2-** **8d and 28°** **C.**

Research results indicated a decline in the reactor efficiency, with increase of organic input load. Mean COD removal efficiency in solid retention times of 2, 4, 6, and 8 days was 82.7, 92.45, 95.97 and 96.1%, respectively. As can be seen in Figure 4, with increase of hydraulic retention time from 0 to 80 minutes, naphthalene removal efficiency increases so as with increase of naphthalene concentration, removal efficiency decreases. In Figure 5 is illustrated the naphthalene removal from water by bacterium pseudomonas aeruginosa. Naphthalene concentration from 20 mg/L came to 5.4 mg/L after lapse of 80 minutes. To verify the amount of naphthalene auto-decomposition, control reactor was used. At length, it was observed that anoxic/aerobic process was about 60% more effective than control reactor in naphthalene removal. Naphthalene removal in retention times of 20, 40, 60 and 80 min was 29%, 50%, 72% and 94%, respectively. In the control reactor, for the same retention times, removal efficiency was 60% lower, which can be attributed to auto-evaporation processes. The system bacterial counting in the beginning and end of this experiment was 6.5 × 10^3^ bacteria per ml and 3.5 × 10^5^ bacteria per ml, respectively. Results of this study indicated that during the anoxic/aerobic process bacterium pseudomonas aeruginosa was able to bring average naphthalene concentration from 20 mg/L to 5–7 mg/L, implying 55–64.3% removal efficiency.

### Effect of solution flow rate on naphthalene degradation during anoxic–aerobic process

**Figure 6:**
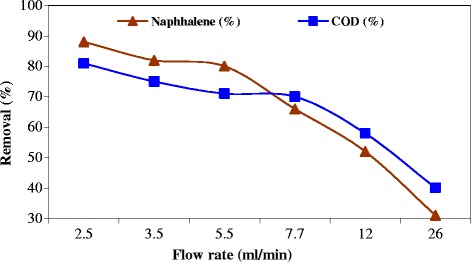
**The biological degradation of naphthalene at the flow rate of 2.5,** **3.5,** **5.5,** **7.7,** **12,** **and 26 mlmin** ^**−1**^ **at pH 8.**

The biological degradation of naphthalene (10 mg/L) was conducted at the flow rate of 2.5, 3.5, 5.5, 7.7, 12, and 26 ml/min. The results are shown in Figure 6. As observed, the efficiency of naphthalene degradation was 88%, 82%, 80%, 66%, 52% and 31% at pH 8, respectively. The naphthalene concentration of each output was kept constant. It takes at least 12 h to completely degrade naphthalene. The residence time of the naphthalene solution was estimated by dividing the volume of the bioreactor (5 L) by the flow rate. The removal of naphthalene and COD decreases with increasing the flow rate, in other words, decreasing the residence time, as reported [14]. The effect is due to the decreasing residence time for microorganisms to contact with naphthalene and COD. Figure 6 also shows that the remained COD concentration becomes higher than naphthalene concentration with decreasing the flow rate of naphthalene solution. The degradation of COD requires a longer time than the degradation of naphthalene. On the other hand, naphthalene evaporation was increase with increasing the flow rate, as can be seen in control reactor (Figure 5). Accordance with this report, previous studies results have indicated that the COD still remained after naphthalene completely degradation [15].

### Effect of solution pH on naphthalene degradation during anoxic–aerobic process

**Figure 7:**
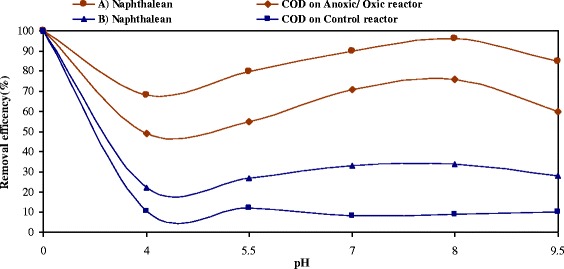
**Naphthalene and COD removal efficiency in vertical anoxic–** **aerobic continuous flow combined bioreactor and control reactor at different pH levels,** **for cellular retention time of 8 days at 28°** **C and flow rate 2.5 mlmin** ^**−1**^ **.**

Among other effective parameters on naphthalene removal, pH level is important. In this study, effect of pH 4, 5.5, 7, 8 and 9.5 on naphthalene removal process was tested. Naphthalene removal efficiency by bacterium pseudomonas at pH 8 was 96% and at pH 4, 5.5, 7 and 9.5, 68, 80, 90 and 85%, respectively. COD removal efficiency at these pHs varied between 68-96%, and with pH increase, the system removal efficiency improved. Other results of the experiment are presented in Figure 7.

### Effect of naphthalene concentration

The artificial wastewater containing the naphthalene, glucose and mineral medium was prepared with the naphthalene concentration of 0.5-20 mg/L and the initial COD 100 ± 18 mg/L. In Table 3, effect of the vertical anoxic–aerobic continuous flow combined bioreactor and control reactor on naphthalene and COD concentration and their removal efficiency is presented.

**Table 3:** **effect of vertical anoxic**–**aerobic continuous flow combined bioreactor and control reactor on naphthalene and COD concentration and their removal efficiency**

**Naphthalene concentration**		**Naphthalene removal efficiency**		**COD**
		**Anoxic/Oxic reactor**	**Control reactor**	
**0.5 mg**/**l**	(%)	66	27	78
	mg/l	0.17 ± 0.6	0.365 ± 0.01	0.11 ± 4.2
**1 mg**/**l**	(%)	58.4	21.5	94
	mg/l	0.416 ± 0.02	0.785 ± 0.05	0.06 ± 0.03
**5 mg**/**l**	(%)	51	26.5	90
	mg/l	2.45 ± 1.5	3.675 ± 0.21	0.5 ± 0.6
**10 mg**/**l**	(%)	44.5	19.5	92
	mg/l	5.55 ± 0.5	8.05 ± 2.15	0.8 ± 0.05
**20 mg**/**l**	(%)	32.5	17	89
	mg/l	13.5 ± 4.50	16.6 ± 5.48	2.2 ± 1.35

### Effect of the turbidity formed by bacterial action on naphthalene removal efficiency

**Figure 8:**
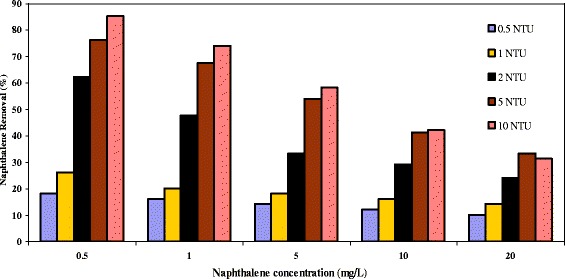
**Naphthalene removal efficiency with produced turbidity by bacterium (in NTU), (cellular retention time of 8 days at 28** **°C**; **solution pH:** **7 and flow rate 2.5 mlmin** ^**−1**^ **).**

In this section, to investigate the effect of bacterium pseudomonas aeruginosa on naphthalene concentration, different turbidity levels produced by the bacterium was examined. Hence, after culture of the respective bacterium in R_2_A environment, a bacterial suspension with 0.5-10 NTU was formed. Next, concentrations of 0.5-20 mg/L in each turbidity level were examined. The results are presented in Figure 8. At 0.5 mg/L concentration naphthalene, a removal efficiency of naphthalene 18-85% was obtained, so as at higher turbidity levels, higher removal efficiency was realized.

Pseudomonas is a gram-negative rod-shaped bacterium with high ability in decomposition of organic and oil pollutants, including naphthalene. Its ability depends on production of catalytic enzymes and formation of metabolic paths [16]. Due to presence of various degrading enzymes in bacteria, they have stronger effect on naphthalene degradation process relative to other microorganisms. In this study, following culture of bacterium pseudomonas aeruginosa and formation of the microbial suspension from it and having accomplished the compatibility process between this microorganism and naphthalene (which is the indicator of aromatic hydrocarbons), they were blended in a combined anoxic/aerobic reactor. As the solid retention time increased from 2 days to 8 days, COD and naphthalene removal efficiency gradually increased. On day 8, removal efficiency of naphthalene and COD reached 94 and 96.1%, respectively. Beklemishev et al. [17] using the extracted bacteria from soil reported a maximum degradation efficiency of 58-73% for 3 and 4-membered ring aromatic compounds after 7 days. Seoud et al. [18] by inseminating bacterium pseudomonas into a biological reactor within 5–7 days managed to achieve a naphthalene removal efficiency of about 60%. Kozlova et al. [19] using pseudomonas putida G7 investigated naphthalene degradation in the soil sample. They succeeded within 7 days to achieve removal efficiency of 95%. On day 4, naphthalene degradation in the soil sample was 85% and after 6 days 90% naphthalene was removed [19]. Feijoo–Siota et al. [20] using pseudomonas stutzeri reported a naphthalene biologic degradation of 93% within 6 days. A naphthalene degradation speed of 0.2 ml/L per hour was obtained by Pathak by injection of flavor bacterium strain in to a biological reactor. In addition, the produced enzymes in this process were identified by him [16].

The obtained results in laboratorial condition indicate feasibility of naphthalene degradation by injection of bacterium pseudomonas into a biological anoxic/aerobic reactor. Although auto-decomposition of such volatile hydrocarbon as naphthalene can also occur at environment temperature, this decomposition in vicinity of pseudomonas in a biological anoxic/aerobic reactor can be enhanced up to several times (in this study up to 60% [21]. During anoxic degradation process, biological metabolites such as diphenylmethane, cyclohexane, hydroxytoluene, and alpha-Cadinol are produced from degrading bacteria, which facilitate degradation process. These compounds, in naphthalene degradation, act like surfactants and effect on solution pH [22].

In addition, bacteria are able to change and adapt themselves to changes in environmental condition such as a change from anoxic to aerobic condition. In either anoxic or aerobic condition, bacterium pseudomonas heterotrophy is able to use naphthalene as the aromatic compounds indicator and as a source of carbon [23]. Change of bacterium metabolism from anoxic to aerobic helps it produce the required enzymes in shorter time. Among anoxic/aerobic advantages, it can be referred to lower utilization cost, less biomass production during anoxic process, and finally less slime decomposition. Lower cost of this method in aromatic hydrocarbons removal such as naphthalene is very beneficial. In nature, aromatic compounds decomposition is influenced by environmental parameters such as pH, temperature degree, and amount of injected bacterium [24].

From ANOVA analysis, effect of the five parameters on naphthalene degradation was examined. The results indicated the statistical significant of the five parameters (p = 0.0023, 0.0078, 0.0064, 0.0011 and < 0.0001), and for COD degradation (p-values are smaller than 0.05). The obtained R^2^ is 0.92 which indicate that 92% of the information results are correct and 6% of these experiments have error (Table 4).

**Table 4:** **One**-**way ANOVA for naphthalene degradation at different pH**, **SRT**, **Flow rate and naphthalene concentration**

**Parameter**	**No. of groups**	**F**	**P**-**value**	**F crit**
pH	5	13.05	0.0023	2.47
Time	4	16.4	0.0078	6.57
Flow rate	6	11.25	0.0064	4.65
Naphthalene concentration	5	11.5	0.0011	3.55
Bacterium injection	5	15.5	0.0001	4.88

R^2^ = 0.92.

From this study and based on numerous studies, it was found that pH control within range of 7–8 could increase the process efficiency [25]. Base pH boundary 7.8-8.5 had the strongest effect on naphthalene degradation. pH can affect microbial activityand therefore investigations into the effect of enzyme activity, transport processes and the nutrient solubility were made. As shown in Figure 7, the highest removal efficacy and lowest remaining concentration of naphthalene was found at pH 8.0. More than 96% of naphthalene and 76 of COD was degraded when the pH of the bioreactor ranged from 7 to 8. Degradation efficiency however significantly declined when the pH was <6.0 or >8.5. These results are consistent with most studies, where microorganisms favoured growth at pH levelsranging from 6.0 to 8.0 [25,26]. It is likely that such acidic or alkalineconditions affect bacterial activity, and hence naphthalene degradation [27].

In addition, the greatest naphthalene degradation was obtained at 25-37°C at constant bacterium injection doses. The amount of bacterium injection into the system was statistically significant (p < 0.0001) and relative to other parameters its effect was found stronger. Increase in dose of bacterium injection into the system is goes along with increase of naphthalene degradation. At pH 8 and 10 NTU which is the greatest amount of bacterium injected to the system, the highest naphthalene removal efficiency was obtained. At lower pH, retention time and injection quantities, a minimum of 63% removal efficiency was obtained, where as at higher pH and retention time (80 min), removal efficiency could be brought to around 100%. The same result obtained from other studies [28].

As the initial concentration of naphthalene increased from 0.5 to 20 mg/L, the remaining concentration of naphthalene decreased from 33.4% to 65.5% after 3 days. The removed amount of naphthalene using strain P. aeruginosa however decreased as the initial concentration of naphthalene increase, which is due to the initial concentration that provides an important driving force to overcome all mass transfer resistances of the naphthalene between the aqueous and solid phases [29]. Consequently a lower initial concentration of naphthalene may enhance the process. This suggests that strain P. aeruginosa could survive and rapidly degrade a high concentration of naphthalene at 20 mg/L. Despite the substrate concentrations being high enough to support growth, this may cause toxic effects, and toxic metabolites may accumulate in the growth medium. However, the tolerated high concentration of naphthalene in the vertical anoxic–aerobic continuous flow combined bioreactor means that strain P. aeruginosa is a potential alternative strategy in naphthalene degradation [29].

## Conclusions

This work highlights the link between the Pseudomonas aeruginosa and the degradation of naphthalene as PAH in a wastewater subjected to anoxic/oxic condition on vertical anoxic–aerobic continuous flow combined bioreactor. A novel Pseudomonas aeruginosa counted and injected on vertical anoxic–aerobic continuous flow combined bioreactor can be used to effectively biodegrade naphthalene. It emerged that the degradation of naphthalene rise to 94% within 8 days under optimum conditions (temperature 27°C, pH 8.0, and naphthalene concentration of 20 mg/L). Naphthalene concentration, retention times, flow rate, pH and turbidity formed by bacterial in the bioreactor have been shown to be of particular importance in the removal of naphthalene. Naphthalene degradation increased with the increasing naphthalene concentration to 20 mg/L. Alkaline pH [7,8] was favorable for Pseudomonas aeruginosa for naphthalene degradation. More than 96% of naphthalene and 76% of COD was degraded when the pH of the bioreactor ranged from 7 to 8. Increase in dose of bacterium injection into the system is goes along with increase of naphthalene degradation. At pH 8 and 10 NTU which is the greatest amount of bacterium injected to the system, the highest naphthalene removal efficiency was obtained. As the initial concentration of naphthalene increased from 0.5 to 20 mg/L, the remaining concentration of naphthalene decreased from 65.5% to 33.4% after 3 days. Based on experimental results, it was determined that this process can effectively reduce naphthalene under optimal conditions and this method can be used for the removal of similar compounds.

## Abbreviations

(PAH): Poly aromatic hydrocarbon
(COD): Chemical oxygen demand
(POM): Polycyclic organic matter
